# Preoperative Plan with 3D Printing in Internal and External Fixation for Complex Tibial Plateau Fractures

**DOI:** 10.1111/os.12466

**Published:** 2019-07-10

**Authors:** Wei‐yong Wu, Wei‐guo Xu, Chun‐you Wan, Min Fang

**Affiliations:** ^1^ Department of Orthopaedics Tianjin Hospital Tianjin China; ^2^ Tianjin Medical University Metabolic Diseases Hospital Tianjin China

**Keywords:** Tibial plateau fracture, 3D printing, Internal fixation, External fixation

## Abstract

**Objective:**

To compare short‐term treatment effects of internal and external fixation in the treatment of complicated tibial plateau fractures by preoperative planning with 3D printing.

**Methods:**

Sixty‐nine patients with tibial plateau fractures were examined. 3D printing was used to establish the model in all patients before the operation. Thirty‐four patients were treated with an external fixator (9‐Schatzker Type V, 25‐Schatzker Type VI) and 35 patients were treated with internal fixation (12‐Schatzker Type V, 23‐Schatzker Type VI). The time span of the study was 2 years after the operation. All patients were followed up in the clinic of the attending physician who recorded patient follow‐up information at the same time. Finally, the Rasmussen functional score, radiographic parameters, complication rates, hospital days and operative parameters of the two groups were analyzed.

**Results:**

The short‐term (within 2 years) Rasmussen score in the external fixation group was close to that of the internal fixation group; the differences were not significant (*P* > 0.05). The fractures were reduced adequately using both forms of surgical treatment. There is no significant difference between internal and external fixation in terms of radiographic parameters after 2 years (Mann–Whitney *U*‐tests, *P* > 0.05). Thrombosis was detected in 7 cases (2 external fixation, 5 internal fixation). Superficial infection was detected in 3 cases (1 external fixation, 2 internal fixation). Deep infection was detected in 3 cases (0 external fixation, 3 internal fixation). Knee stiffness was detected in 4 cases (2 external fixation, 2 internal fixation); 1 (2.7%) case of screw pullout occurred in the internal fixation group. The external fixation group had shorter operation times (172.94 ± 50.00 min *vs* 253.86 ± 64.59 min), less bleeding volume (395.88 ± 121.10 mL *vs* 864.29 ± 238.12 mL), and fewer days (17.03 ± 5.03 days *vs* 30.17 ± 8.64 days) of hospitalization compared to the internal fixation (*t*‐test, *P* = 0.00); subgroup analysis of all patients with complex tibial plateau fractures revealed that for patients with tibial plateau fracture type VI, the functional score of external fixation (26.79 ± 2.04) is better than that (25.54 ± 1.69) of internal fixation (*t*‐ test, *P* = 0.026) and the overall infection rate of external fixation is lower than that of internal fixation (χ^2^‐ test, *P* = 0.047).

**Conclusion:**

Using 3D printed models in combination with external fixation has more advantages for short‐term treatment of complex tibial plateau fractures. In particular, relatively better functional recovery and lower rates of infection can be achieved for Schatzker type VI fractures. The external fixation treatment was preferred in cases of Schatzker VI tibial plateau fractures.

## Introduction

The tibial plateau fractures represent 1%–2% of all fractures and approximately 8% of fractures in the elderly^1^. For assessment of the initial injury, orthopaedic surgeons widely use the Schatzker classification to observe the initial fracture morphology, to plan the surgical method, and to evaluate the prognosis of the operation, which divides fractures into six different groups: Schatzker I to Schatzker VI^2^, ^3^. Of these, types V and VI are considered as complex tibial plateau fractures. Schatzker type V and VI are high‐energy tibial plateau fractures often associated with extensive soft tissue injury and complex intra‐articular injuries, which can influence surgical treatment^4^, ^5^, ^6^. Postoperative complications such as wound healing problems, infections, and secondary degenerative arthritis are quite common. They tend to have a poor prognosis. High energy complex fractures of the tibial plateau remain a challenge to even the most experienced surgeons^7^. The goals of treatment in such high‐energy tibial plateau fractures are to maintain joint stability, congruity, and alignment without much soft tissue dissection, thereby helping in early mobilization of the knee joint^8^. This requires a surgeon to have a clear understanding of the fracture morphology and to choose a reasonable fixation method according to the fracture morphology. Detailed preoperative planning, correct choice of operation, and selection of fixation methods have a very important impact on patients’ prognosis.

In the current medical literature, there is no consensus about which approach is preferable for treating these fractures. The best treatment method remains controversial^9^. Treatment methods for high‐energy tibial plateau fractures include definitive external fixation, dual plating, and intramedullary nailing, but plating internal fixation and hybrid external fixation are the two most commonly used methods^10^. Both surgical methods have their pros and cons. Open reduction and internal fixation of individual tibial plateau helps maintain anatomic articular congruity and restoration of mechanical alignment and allows early mobilization of the knee joint. However, extensive exposure of the lateral and medial side is often required; the combination of original injury damage with the extensive surgical approach led to a high rate of complications, including wound healing problems and infection^11^. External fixation (EF) represents a valid alternative method because of its easy application and minimal surgical exposure. It may also require a shorter hospital stay with its attendant benefits. Problems with these techniques include the inconvenience of an external apparatus that requires careful maintenance, and the possibility of pin tract infection and subsequent collapse with lack of reduction of the fragments. The use of external fixators as a mode of treatment also leads to joint stiffness because of delayed mobilization of the knee joint.

Tibial plateau fractures (Schatzker type V and VI) are often accompanied by the collapse of the articular surface and mutual occlusion of the fracture block. Hence, recognition and comprehension of the fracture features will help orthopaedic surgeons to understand the injury mechanism better and to manage these fractures by planning optimal surgical procedures. Current surgical approaches to tibial plateau fractures rely on X‐ray films and CT images, which can be limited in their utility for understanding the fracture and can increase the difficulty and risk of the operation. Because an anteroposterior radiograph cannot be used to evaluate posteromedial and posterolateral shear fractures^12^, controversy exists in the literature as to whether routine CT evaluation can contribute towards changes in preoperative planning in comparison with plain radiographs^13^, ^14^, ^15^. 3D printing technology allows clinicians to accurately design surgical approaches for fracture treatment to allow for more accurate surgical outcomes and to improve the quality of reduction and operational reliability. Thanks to 3D printing technology, the fracture morphology can be recreated as a physical model^16^, allowing clinicians to observe the degree of tibial plateau injury and the planar fracture collapse position. 3D modeling can allow surgeons to observe fracture morphology and collapse more intuitively, to design more accurate surgical approaches and reduction methods for internal fixation operation, which can avoid unnecessary soft tissue stripping and reduce wound complications. It can also help external fixation to make up for the defect of not looking directly at the articular surface, improve the reduction quality of external fixation, and rationally plan the position of tibial half‐pins. Previous studies have taken into account the impact of different surgical procedures on tibial plateau fractures but have not considered the impact of reasonable preoperative planning with 3D printing on different fixation methods.

In this experiment, interventions were extended to preoperative planning to provide patients with more comprehensive treatment options. The objectives were: (i) to observe the effects of two different fixation methods on complex tibial plateau fractures by combining preoperative 3D printing; (ii) to provide more effective surgical methods and fixation methods for complex tibial plateau fractures; and (iii) to analyze the advantages and disadvantages of internal fixation and external fixation.

## Materials and Methods

### *Inclusion and Exclusion Criteria*

Inclusion criteria were as follows: (i) patient or population: patients with complex tibial plateau fractures (according to Schatzker classification V, VI); (ii) intervention/exposure: treated using a hybrid external fixation; (iii) comparison/control: open reduction and internal fixation; (iv) outcome: ① knee joint functional evaluation, ② radiographic parameters, ③ incidence of complications, and ④ operative parameters and hospital days. Exclusion criteria were as follows: (i) Patient were with open fracture; (ii) Patient has not undergone surgery for more than three weeks; (iii) patients were with pathological fracture; (iv) patients were with neurological and vascular injuries; and (v) patients were with with a history of arthritis.

### *Basic Information*

We examined a consecutive series of 69 patients with complex tibial plateau fractures admitted to the trauma department at Tianjin Hospital between November 2014 and June 2016. Sixty‐nine patients met the criteria and were divided into two groups based on the fixation method (internal *vs* external). There was no significant difference between the two groups in age, gender, or Schatzker classification (*P* > 0.05) (Table 1).

**Table 1:** Group 2 basic information comparison

Groups	Number	Gender (cases)		Age (years, mean±SD)	Schatzker type (cases)	
		Male	Female		V	VI
Internal fixation	35	25	10	48.51 ± 9.98	12	23
External fixation	34	21	13	48.56 ± 11.67	9	25
Statistic value		χ^2^ = 0.725		*t* = −0.017	χ^2^ = 0.03	
*P* value		0.395		0.986	0.856	

### *Establishment of Digital 3D Model*

The equipment used dual‐source 64‐slice spiral CT (Siemens) from the Radiation Center of Tianjin Hospital to scan the proximal tibia with a 1‐mm thin slice. After scanning, the data were input into M3D Medgraphic Digital Medical Software (Shanghai Jiao tong University Laboratory) in Dicom format. The proximal tibia 3D model was reconstructed in the M3D digital medical software. The related data were measured and the fracture reduction was simulated at the same time. Makerbot software (Designed in Brooklyn, NY USA) is used to transform STL files into the file with a .×3g suffix. Using the HY‐500 3D printer (Nanjing Songshang Medical) and ABS material (Hangzhou Xusheng New Material), the exact same fracture model in a 1:1 ratio was printed out.

### *Surgical Methods*

Preoperative preparation, anesthesia methodology during surgery, and the surgeons were kept consistent between the two groups.

**Figure 1:**
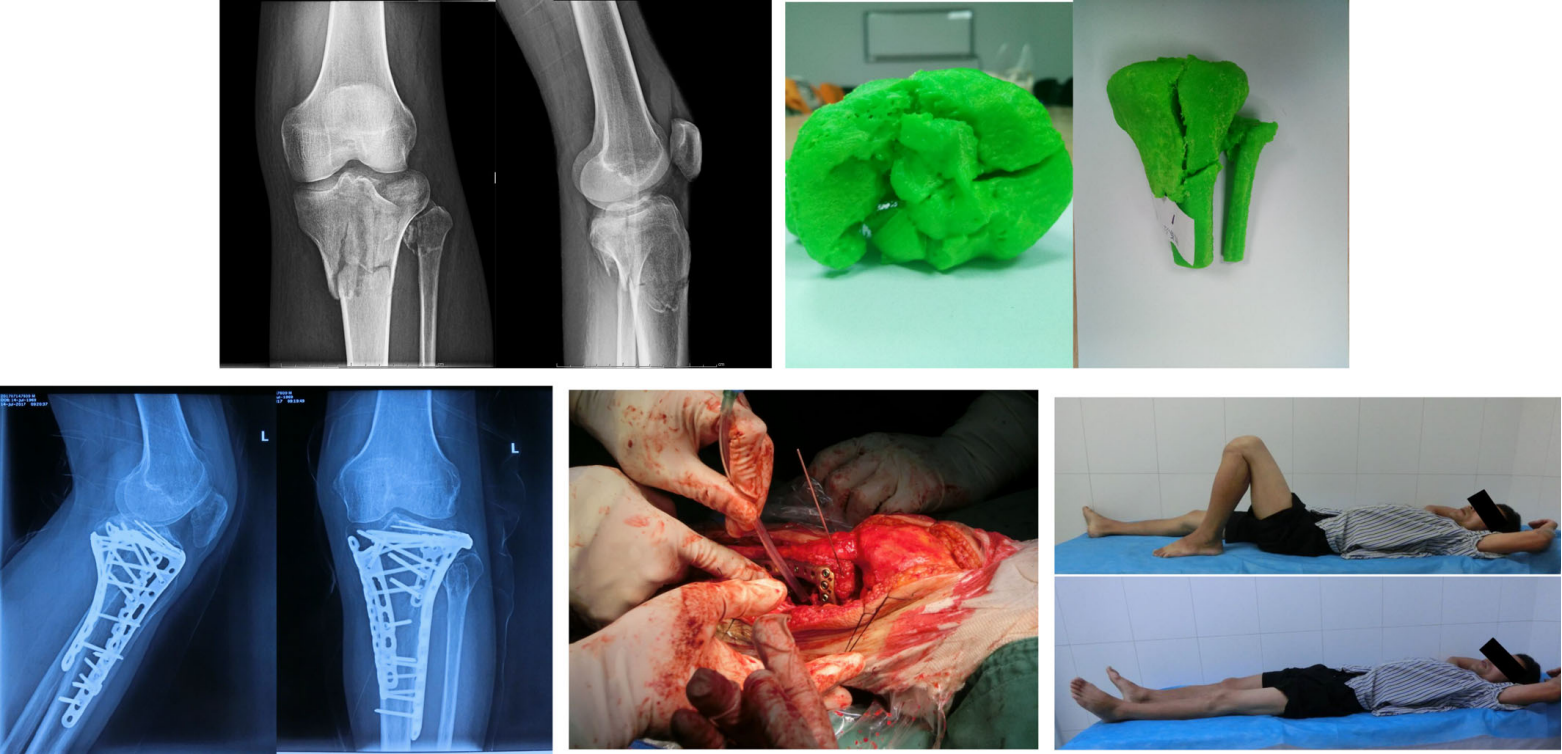
Male patient, 50 years old. A traffic accident resulted in type VI fracture of the tibial plateau. During surgery, comminuted fracture of articular surface and proximal tibia fracture were found. The articular surface was reduced and fixed with a locking plate. The knee joint function was good 2 years after surgery.

**Figure 2:**
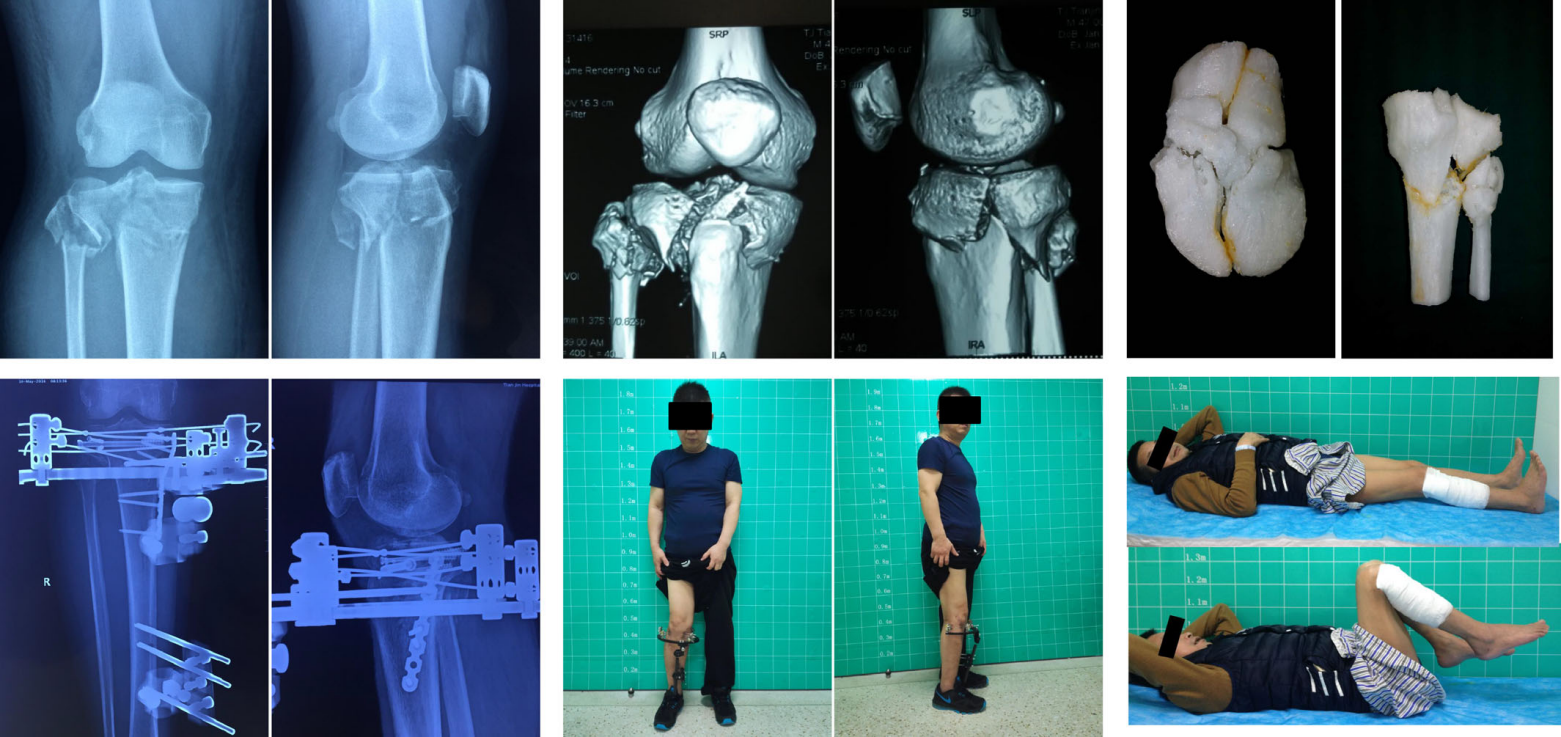
Male patient, 45 years old. Fracture of Schatzker type VI tibial plateau caused by traffic accident. Splitting and collapse of the articular surface were found during the operation. External fixation combined with limited internal fixation maintained the stability of the articular surface. After 3 months, the external fixator was removed. The knee joint function was good 2 years after surgery.

**Figure 3:**
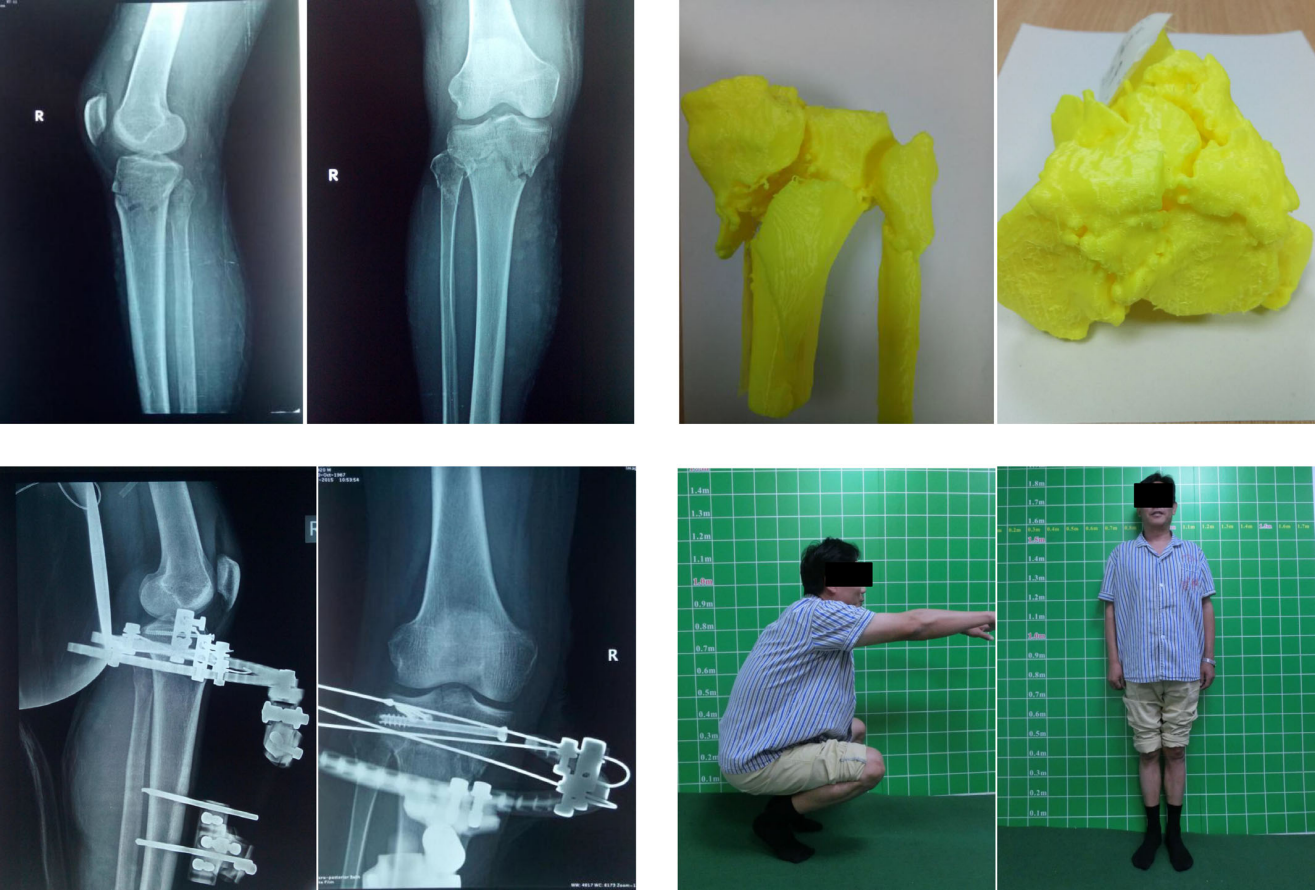
Male patient, 47 years old. Following traumatic right knee local swelling, pain, deformity, and movement limitation, the patient was admitted to hospital for 4 hours. Under combined spinal–epidural anesthesia, right tibial plateau fracture was treated with prying reduction, hollow screw fixation, and external fixator fixation. After 2 years’ follow‐up, the function was good.

In the internal fixation group, the principle of exposure and fixation of the AO principle is used for the single anterior incision or the combined medial and lateral incision according to the specific type of fracture. The joint was opened and lag screws were used for reduction and fixation of the articular surface. Plates were applied in every knee medially and laterally to reestablish tibial alignment and to buttress the articular repair. Intraosseous implantation of Wright artificial bone followed. Standard wound closure over drains was performed (Typical cases are shown in Fig. 1). In the external fixation group, the patients were managed with limited open reduction of the articular surface followed by the insertion of percutaneous lag screws or plating to stabilize. A small incision over the antero‐medial portion of the tibia metaphysis was made in the tibial cortex. The periosteal dissector was used to perform the prying reduction of the fracture block under fluoroscopy. After the bone fragments were precisely reducted the hybrid external fixators is applied. The frame consisted of a proximal ring with four K‐wires (two with olives and two without) and three distal self‐tapping cortical pins. Two connecting rods were used to complete the frame (Typical cases are shown in Figs 2,3).

### *Evaluation Criteria*

#### *Radiographic Parameters*

The radiographic parameters^17^: Reduction was recorded as anatomical or with displacement of 1 mm to 2 mm or 3 mm to 4 mm. Collapse was recorded as 0–2 mm, 2–4 mm, or ≥4 mm. Alignment was recorded as anatomical or with angulation between the metaphysis and the diaphysis of 0° to 5° or ≥5°. All radiographic parameters were measured by CAD 2007 (Computer Aided Design, Autodesk, USA).

#### *Rasmussen Knee Clinical Functional Score*

The Rasmussen knee clinical functional score^18^ was used to evaluate the functional recovery of the knee. Rasmussen's total score of knee joint function for tibial condylar fracture was 30; 20 or more points was considered satisfactory or excellent, and less than 20 points was considered unsatisfactory. Rasmussen functional score were scored by patients in the outpatient clinic according to the recovery situation.

#### *Postoperative Infection*

The main result was infection. Infection can be divided into superficial infection and deep infection. Superficial infection was defined as wound drainage requiring oral antibiotic treatment and/or any suppuration found on the fascial surface. Deep infection is defined as any purulent wound deep into the fascia or requiring multiple debridements.

### *Statistical Analysis*

All data were analyzed with IBM SPSS version 23.0 (IBM, Armonk, NY, USA). First, the Shapiro–Wilk test is used to determine whether the measurement data is normally distributed. The patients’ age, operation time, hospitalization days, bleeding volume, and Rasmussen's functional score were normal distribution data, and the variance was homogeneous. The independent‐samples *t*‐test was used for analysis. The χ^2^‐test was used to analyze the difference in the age, Schatzker classification, complications, and other counting data between the two groups. Mann–Whitney *U*‐tests were used to analyze the radiograph parameters. The comparison of Rasmussen's functional score at different time points in the same group was made by variance analysis of repeated measurements. If the difference was significant, then LSD‐*t* test was used to compare the two measurements at different time points. P<0.05 indicated that there was significant difference between the two measurements.

### *Follow‐up Contents*

All patients were followed up in the clinic of the attending physician from their definitive fixation. All patients were routinely reexamined; X‐rays were performed and functional scores were recorded at the same time. Follow‐up and information collection were performed 3 months, 6 months, and 2 years after surgery.

## Results

### *Function and Quality of Reduction*

The short‐term (within 2 years) Rasmussen score in the external fixation group was close to that in the internal fixations group; the differences were not significantly different (*P* > 0.05). The fractures were reduced adequately using both forms of surgical treatment. There is no significantly difference between internal and external fixation in terms of radiographic parameters after 2 years (Mann–Whitney *U*‐test, *P* > 0.05). Tables 2 and 3.

**Table 2:** Functional score comparison (mean±SD)

Groups	Preoperation	Three months after operation	Six months after operation†	Two years after operation
Internal fixation	5.91 ± 1.38	17.46 ± 1.91*	20.74 ± 1.52* †	25.41 ± 2.67* † ‡
External fixation	5.68 ± 1.70	16.53 ± 2.22*	21.88 ± 1.22* †	26.09 ± 2.76*† ‡
Statistic value	*t* = 1.861	*t* = −0.419	*t* = 0.639	*t* = −1.019
*P* value	0.525	0.067	0.677	0.312

* In the same group, compared with preoperation, *P* < 0.05.

† In the same group, compared with 3 months after the operation, *P* < 0.05.

‡ In the same group, compared with 6 months after the operation, *P* < 0.05

**Table 3:** Imaging parameters

Radiographic parameters	Internal fixation	External fixation
	2 year (preoperative)	2 year (preoperative)
Joint reduction achieved (*P* = 0.538)		
Anatomical	13 (0)	11(0)
Displacement 1–2 mm	16 (0)	15 (0)
Displacement 3–4 mm	6 (35)	8 (34)
Metaphyseal–diaphyseal alignment (*P* = 0.818)		
Anatomical	19 (0)	20 (0)
Angulation 0° to 5°	15 (16)	13 (16)
Angulation >5°	1 (19)	1 (18)
Joint collapse (*P* = 0.487)		
Joint collapse 0–2 mm	25 (0)	21 (0)
Joint collapse 2–4 mm	7 (0)	11 (0)
Joint collapse >4 mm	3 (35)	2 (34)

### *Complications*

Thrombosis was detected in 7 cases (2 external fixations; 5 internal fixations). The difference was not significant between two groups (χ^2^‐test, *P* = 0.449). Superficial infection was detected in 3 cases (1 external fixations; 2 internal fixations) deep infection was detected in 3 cases (0 external fixation; 3 internal fixation). This difference was not significant between the two groups (χ^2^‐test, *P* = 0.239). Knee stiffness was detected in 4 cases (2 external fixations; 2 internal fixations) requiring manipulation under anesthesia; 1 (2.7%) case of screw pullout occurred in the internal fixation group. See Table 4.

**Table 4:** Compared rate of complications among different groups of fracture (cases[%])

Complications	Internal fixation	External fixation	*P* value
Thromboembolism incidence	5 (14.2)	2 (5.8)	0.449
Superficial infection	2 (5.7)	1 (2.9)	1
Deep infection	3 (8.5)	0	0.239
Knee stiffness	2 (5.7)	2 (5.8)	1
Screw pullout	1 (2.7)	0	1

### *Operative Parameters and Hospital Days*

The external fixation group had shorter operation times (172.94 ± 50.00 min *vs* 253.86 ± 64.59 min), less bleeding volume (395.88 ± 121.10 mL *vs* 864.29 ± 238.12 mL), and fewer days (17.03 ± 5.03 days *vs* 30.17 ± 8.64 days) of hospitalization compared to the internal fixation group (*t*‐test, *P* = 0.00; Table 5).

**Table 5:** Operative parameters and hospital days (mean±SD)

Groups	Number	Operation time (min)	Bleeding volume (mL)	Days in hospital (day)
Internal fixation	35	253.86 ± 64.59	864.29 ± 238.12	30.17 ± 8.64
External fixation	34	172.94 ± 50.00	395.85 ± 121.10	17.03 ± 5.03
*t* value	—	5.807	10.252	7.192
*P* value	—	0	0	0

### *Subgroup Analysis*

Subgroup analysis of all patients with complex tibial plateau fractures revealed that the two fixation methods resulted in functionally similar outcomes (25.55±4.03 *vs* 24.40±3.57) for patients with Schatzker V tibial plateau fracture 2 years later (*t*‐test, *P* = 0.501). Only 1 patient with external fixation had superficial infection among all patients with Schatzker V plateau fractures. There was not significant difference between internal fixation and external fixation in terms of infection rate (χ^2^‐test, *P* = 0.501; Table 6 ①).

**Table 6:** Subgroup analysis

Group	Rasmussen functional score	Superficial infection	Deep infection	Overall infection
➀ Schatzker type V				
Internal fixation (*n*=12)	25.55 ± 4.03	—	—	—
External fixation (*n*=9)	24.40 ± 3.57	1(11%)	—	11%
Statistic value	*t* = 0.686			Fisher exact text
*P* value	0.501			0.429
➁ Schatzker type VI				
Internal fixation (*n*=23)	25.54 ± 1.69	2(8.6%)	3(13%)	5(21.7%)
External fixation (*n*=25)	26.79 ± 2.04	—	—	0%
Statistic value	*t* = −2.308			χ^2^ = 3.96
*P* value	0.026			0.047

For patients with tibial plateau fracture of Schatzker VI, the functional score of external fixation (26.79 ± 2.04) was better than that (25.54 ± 1.69) of the internal fixation patients 2 years later (*t*‐test, *P* = 0.026). The overall infection rate of external fixation is lower than that of internal fixation (χ^2^‐test, *P* = 0.047; Table 6 ②).

## Discussion

Complex tibial plateau fractures are intra‐articular fractures caused by high‐energy damage. Surgical treatment for these types of fractures aims to maintain the normal force lines of the lower extremities to restore the anatomic reconstruction of the articular surface and allow early knee mobilization. The long‐term target of treatment is full recovery of knee joint function^19^. Indications for surgery are the collapse and separation of the articular surface >2 mm, angular deformity of >10° in the coronal (varus‐valgus) or sagittal plane, open vascular injury, and osteonecrosis syndrome^20^. The quality of the reduction of the tibial plateau fracture and the choice of surgical method has a direct impact on the functional recovery of the knee joint. Given this, it is very important to have a full understanding of the shape of the fracture and to choose a suitable surgical method for the operation. Using 3D printing models can aid in gaining a better understanding of the specific condition and classification of the fracture, determining the possible difficulties during surgery^21^. With this additional information, the surgical approach can be customized and the reduction planned according to the fracture type. Using this approach, clinicians can simulate the reduction and fixation process prior to the operation and refer to the model at any time during surgery. This allows the fracture area to be treated more accurately, thereby avoiding unnecessary surgical trauma, shortening the operation time, and reducing complications^22^. There is currently no comparison in the literature for internal fixation and external fixation for the treatment of complex tibial plateau fractures by preoperative plan with 3D printing. This study aims to add to this body of research.

There are numerous proposed approaches for the treatment of tibial plateau fractures^23^, ^24^, ^25^, ^26^; however there is no consensus on an optimal treatment of complex fractures^17^. The most common surgical approaches at present include open reduction and internal fixation and external fixators (whether or not limited internal fixation). The ultimate goal of surgical treatment is to restore the function of the knee joint. Ariffin *et al*.^23^ used external fixators to treat 33 patients with Schatzker type V and VI fractures with 90% of patients achieving good Rasmussen scores. Rademakers *et al*.^27^ followed up for more than 5 years in 119 patients with complex tibial plateau fractures and showed better functional scores after using internal fixation. Ahearn *et al*.^17^ and Jansen *et al*.^24^ demonstrated that the two fixation methods (internal *vs* external) result in functionally similar outcomes. The Canadian Orthopaedic Trauma Society (COTS)^28^ noted that 6 months after surgery, the external fixation group had higher functional score than the internal fixation group; however, similar results are obtained after 1 year. In our study, 3D printing technology was used to compare knee joint function scores after treatment with internal or external fixation. It was concluded that the short‐term (within 2 years) Rasmussen score in the external fixation group was close to the internal fixation group. There was no significant difference between the two groups (*P* > 0.05). This suggests that in terms of function, after combining 3D printing, both operative methods can achieve satisfactory outcomes. However, after subgroup analysis, we found that for the patients with Schatzker VI tibial plateau fractures, the Rasmussen score of the external fixation group 2 years later was (26.79 ± 2.04) better than that of the internal fixation group (25.54 ± 1.69) (*P* = 0.026). This shows that the combination of 3D printing and external fixation has more advantages in treating Schatzker type VI tibial plateau fractures.

The quality of reduction also affects the choice of surgical methods. Goetz *et al*.^29^ suggests that an accurate intra‐articular reduction can result in improved knee joint function postoperatively. Decreasing articular step‐off heights is an essential part of the treatment of complex tibial plateau fractures. Poor reduction of the articular cartilage surface can cause adverse effects such as traumatic arthritis^30^. However, Waston *et al*.^31^ suggests that it is more important to maintain the force line during the treatment of tibial plateau fractures. Although external fixation cannot guarantee a smooth articular surface after reduction, if the whole force line of the lower limbs can be maintained, good results can still be achieved^32^. However, studies by Krupp *et al*.^25^ have demonstrated that external fixation is more likely to cause malunion (7% *vs* 40%) compared to internal fixation. In our study, the fractures were reduced adequately using both forms of surgical treatment. In contrast with Krupp^25^, we found no association between the quality of the reduction and the methods of fixation. Satisfactory reduction quality was achieved using both surgical methods. The result of our experiment is better than that without 3D printing^17^. Using 3D printing technology can remedy the limitations of viewing the surface of the joint in external fixation, to achieve reduction quality similar to internal fixation. The collapsed bone is compressed into the cancellous bone at the metaphyseal end. It is difficult to maintain the quality of articular surface restoration. A biomechanical study also shows that the mechanical strength of complex tibial plateau fractures can be obtained by either internal or external fixation^33^.

Complex tibial plateau fractures can be accompanied by varying degrees of soft tissue injury, and the method of fixation is important for effective control of postoperative complications. Orthopaedic surgeons often use this variable to support the choice of EF and internal fixation. In the case of poor soft tissue, external fixation does not require dissection of a large amount of soft tissue, thus minimizing potential complications. Previous literature shows a low rate of deep infection for external fixation^26^. Bicondylar tibial plateau fractures treated with internal fixation resulted in a higher percentage of major complications, including deep infection and non‐union^34^. However, Some researchers^35^ found that the deep infection rate was lower in internal fixation compared to external fixation. Sciadini and Sims^30^ suggests careful observation of the patient's condition before the operation and minimal dissection of the soft tissue during the operation can reduce associated complications. In our study, we also compared the complications, especially the differences in infection between the two groups. In our series, 3 cases of superficial infection were detected, (1 EF; 2 internal fixation); all cases were successfully cured with local therapy and oral antibiotics. We observed 3 (8.5%) cases of deep infection in the internal fixation group, and no cases in the EF group; our overall superficial infection rate was 4.3%. Our deep infection rate was 4.3%. Our overall rates of infection compared favorably with those of other studies, where a 15.2% rate of superficial infection and 9.6% rate of deep infection have been reported^36^. It is indicated that the preoperative planning of 3D printing technology may reduce the total infection rate. Subgroup analysis showed that the total infection rate of internal fixation was 21.7% and that of external fixation was 0% in Schatzker VI plateau fractures (*P* = 0.047). This indicates that after 3D printing, external fixation can better avoid postoperative infection compared with internal fixation. Using a 3D model can avoid unnecessary surgical trauma and control the infection rate. In the present study, there was no increased infection rate in the external fixation group despite the communication between the Kirschner pins (half in soft tissue, half exposed outside and connected to external fixators) and the outside world. Furthermore, given that the external fixation reduced the pressure on the soft tissue, there was satisfactory wound healing. Thrombosis is another complication of tibial plateau fractures. The rate of thrombosis in the internal fixation group (14.2%) was higher than that in the external fixation group (5.8%). Therefore, thrombosis can result from extended hospital bed stays; the time of hospitalization should be shortened as much as possible.

The external fixation group had less operation time, less bleeding volume, and fewer days of hospitalization compared to the internal fixation group (*P* < 0.05). Morgan suggests the increase in days of hospitalization in patients with internal fixation may have adverse effects for elderly patients and must be considered carefully.^37^ In a comparative study of internal and external fixation, Conserva *et al*.^38^ found that the operation time, bleeding volume, and hospitalization days in the internal fixation group were significantly higher than those in the external fixation group, supporting the results of our study. This suggests that 3D printed models may not eliminate the disadvantages of internal fixation compared with external fixation. One disadvantage of external fixation is that the external fixator must be maintained until the fracture is healed, which can be difficult for patients.

Using 3D printed models in tibial plateau fractures preoperatively can effectively eliminate the disadvantages of limited joint visualization in external fixation treatment and improve the quality of reduction. Combined with 3D printing, external fixation for complex tibial plateau fractures can effectively reduce intraoperative bleeding, intraoperative operation time and hospitalization days, and ensure satisfactory reduction quality and postoperative function. Especially for patients with Schatzker VI fractures, external fixation can effectively avoid the occurrence of complications and achieve better functional results compared with internal fixation. These results are particularly important if we consider that the external fixation treatment was preferred in cases of Schatzker VI tibial plateau fractures. However, there are also deficiencies in external fixation, such as pin tract pain when patients wear external fixators. Other factors should be considered, such as patient age and physical condition, knee function requirements, soft tissue condition, and work needs when choosing the surgical approach.

There are some limitations to this study. The sample size was small and the follow‐up time was relatively short, meaning that only short‐term results could be evaluated. For OA statistics, a longer follow‐up period is needed. None of the patients had a CT examination 2 year later and the related imaging parameters were measured using X‐ray, which may have resulted in errors. The secretions of infected wounds have not been cultured for bacterial culture. This study did not research results of staged treatment using a temporary external fixator.
